# A 2-year follow-up analysis of individuals with internet use disorders treated with the webcam-based telemedicine OMPRIS intervention

**DOI:** 10.1038/s41598-025-12401-z

**Published:** 2025-07-31

**Authors:** Jan Dieris-Hirche, Sebastian Nikofor, Magdalena Pape, Laura Bottel, Martin Diers, Bert Theodor te Wildt, Klaus Wölfling, Peter Henningsen, Anja Neumann, Rainer Beckers, Stephan Herpertz, Nina Timmesfeld

**Affiliations:** 1 Department of Psychosomatic Medicine and Psychotherapy, LWL-University Hospital, Ruhr University Bochum, Bochum, Germany; 2 Psychosomatic Hospital Diessen Monastery, Diessen, Germany; 3 Department of Psychosomatic Medicine and Psychotherapy, Outpatient Clinic for Behavioral Addictions, University Medical Center of the Johannes Gutenberg-University Mainz, Mainz, Germany; 4 Department of Psychosomatic Medicine and Psychotherapy, University Hospital Rechts der Isar, Technical University Munich, Munich, Germany; 5 Institute for Health Care Management and Research, University Duisburg- Essen, Essen, Germany; 6 Competence Centre of Healthcare Telematics, Hagen, Germany; 7 Department of Medical Informatics, Biometry and Epidemiology, Ruhr University Bochum, Bochum, Germany; 8 Department of Clinical Psychology and Psychotherapy, University of Bamberg, Bamberg, Germany

**Keywords:** Internet use disorder, Gaming disorder, Online therapy, Telemedicine, Long-term, 2-year follow-up, Human behaviour, Outcomes research, Patient education

## Abstract

Evidence-based online treatments for internet use disorders (IUD) are poorly studied. The online-based, therapist-guided, motivational intervention program for internet use disorder (OMPRIS) has shown significant effects on reducing IUD symptoms, while the symptoms remained high in the waiting control group. The current study presents a 2-year follow-up analysis of the IUD symptoms. Participants in the OMPRIS study were followed up 2 years after the intervention. The primary outcome was a reduction in IUD symptoms. Secondary outcomes were time spent on the internet, depression and anxiety symptoms, and general life satisfaction. Linear mixed models (LMMs) were performed to assess the outcomes over time across the intervention and three follow-up measurement, controlling for the covariates age and gender. A total of 116 out of 180 OMPRIS participants (64.4%) were reached for the follow-up survey after 2 years. The LMM showed no significant increase in IUD symptom severity across all three follow-up measurements relative to post treatment measurements. Age and gender had no significant influence in the model. Similar results were found for all secondary outcomes. According to this study, IUD can be effectively treated via webcam and is therefore suitable for patients who are unable or unwilling to undergo face-to-face psychotherapy.

**Supplementary Information:**

The online version contains supplementary material available at 10.1038/s41598-025-12401-z.

## Introduction

Internet use disorder (IUD) refers to the uncontrolled and excessive use of different internet applications as a behavioral addiction. Multiple neural pathways underlie human additive behaviors like IUD, whose mechanisms remain incompletely understood^1^. A subtype of IUD is excessive gaming as the first officially recognized subtype, as well as non-gaming internet activities, such as online shopping, pornography use, social networking, and general internet use^2^. IUD prevalence has increased in recent decades, with rates between 6% and 8% around the world, lower in northern and western Europe and higher in Asia and the Middle East^3–5^. The prevalence of IUDs in German populations ranges between 1.2% and 3.1% in older studies^6–8^. Both internet use and the prevalence of IUD have increased as a result of the COVID-19 pandemic^9–12^. A representative German cohort study with a total of 7 survey waves to date investigated the pathological use of computer games and other IUDs in children and adolescents between the ages of 10 and 17. The prevalence of pathological computer game use showed a value of 2.7% before the Covid-19 pandemic (2019), an increase to 6.3% in 2022 and 4.3% in 2023 and a slight decrease to currently 3.6% in 2025^13^.

While there is evidence that cognitive behavioral therapy works well for IUDs^14–17^, there is still a need for new preventative and therapeutic approaches that work better with current treatment^18^. The effectiveness of digital health and eHealth interventions for patients with behavioral addictions is still poorly studied. A systematic review from 2016 found a total of 16 studies testing internet-related interventions in mostly substance addictions (11 studies in smoking, drinking, and opioid abuse) and a few behavioral addictions (5 studies in pathological gambling). Although only 5 of the 16 studies reported effect sizes (*d* = 0.83–1.72), all studies reported positive treatment outcomes for their respective addictive behaviors^19^. Currently, there is only a very limited number of studies examining the use of eHealth interventions specifically for IUD patients. Two large systematic reviews, one in 2016 and one in 2022, each found only a few (pilot) studies that looked at different eHealth approaches for IUD patients. These included cognitive bias modification, virtual reality exposure, and web-based self-help^20,21^.

Based on a pilot study, our research unit conducted the first high-level, multicentre, prospective, single-blinded, randomised controlled trial between 2019 and 2022, testing a webcam-based online therapy for IUD patients (OMPRIS study) being at least 16 years old^22,23^. Post-treatment analysis showed that OMPRIS participants had a significantly greater IUD symptom reduction compared with the controls from baseline to post-treatment with an effect size of *d* = 0.92. The symptom burden of the waiting group remained high and showed no significant spontaneous remission during the observation period. In addition, the short-term follow-up measurements showed that IUD symptom severity remained low even after 6 weeks and 6 months^22^. Health Economic Evaluation of the OMPRIS intervention revealed an improvement of IUD symptoms at moderate additional costs^24^.

Well-conducted randomized clinical trials remain the gold standard for generating estimates of treatment efficacy. However, the true therapeutic value of medical treatment is revealed in the long-term effect^25^. Follow-up times may be limited by cost and logistical considerations. However, a long-term follow-up is essential to quantify the quality of studies and long-term effects^25^. The aim of this follow-up study is therefore to assess the severity of IUD symptoms and psychological distress in patients two years after their participation in OMPRIS. Furthermore, possible factors influencing the results after two years should be analyzed.

## Methods

### Study design of the OMPRIS study

The OMPRIS main study was a multicenter, prospective, single-blinded, randomized controlled trial and was conducted with therapists from four IUD specialized medical centers in Germany^22^. The OMPRIS intervention was developed in a way that it can be used for prevention and therapy. Therefore, the inclusion criteria for OMPRIS were chosen to allow participants with mild symptoms to take part in the intervention. The distribution of symptom severity has been published elsewhere^22^. Recruitment, diagnostics, and intervention were carried out completely online. The OMPRIS intervention group was compared with a waiting control group. Participants took part in a short-term, webcam-based, telemedicine treatment twice a week. In total, participants underwent 4 weeks of telemedicine intervention with at least 2 psychotherapeutic sessions per week and 1 or 2 social counselling sessions over the course of the treatment (approximately 60 min per session). The OMPRIS intervention was manualized and provided therapeutic guidance regarding the adaptations for the webcam-based application^26^. The manual included techniques from motivational interviewing, cognitive behavioural therapy, and acceptance and commitment therapy. These have been shown to be effective at improving health behaviors in people with behavioral addictions, such as IUD^16^.

Additionally, social counselling was offered. Participants were automatically assigned either to the OMRPIS intervention (*n* = 89) or the waitlist control group (*n* = 91). The waiting time for the control group was 4 weeks, which was exactly the duration of the OMPRIS intervention. For ethical reasons, the waiting group also received the OMPRIS intervention after the waiting period. The effects of OMPRIS therapy on the IUD symptom severity were assessed after treatment and after 6 weeks and 6 months^22^. We have published the study OMPRIS protocol, the OMPRIS study manual, and the main treatment effects^22,27,28^. The current study represents the 2-year follow-up assessment.

### Participants recruitment and procedure of the current study

**Fig. 1:**
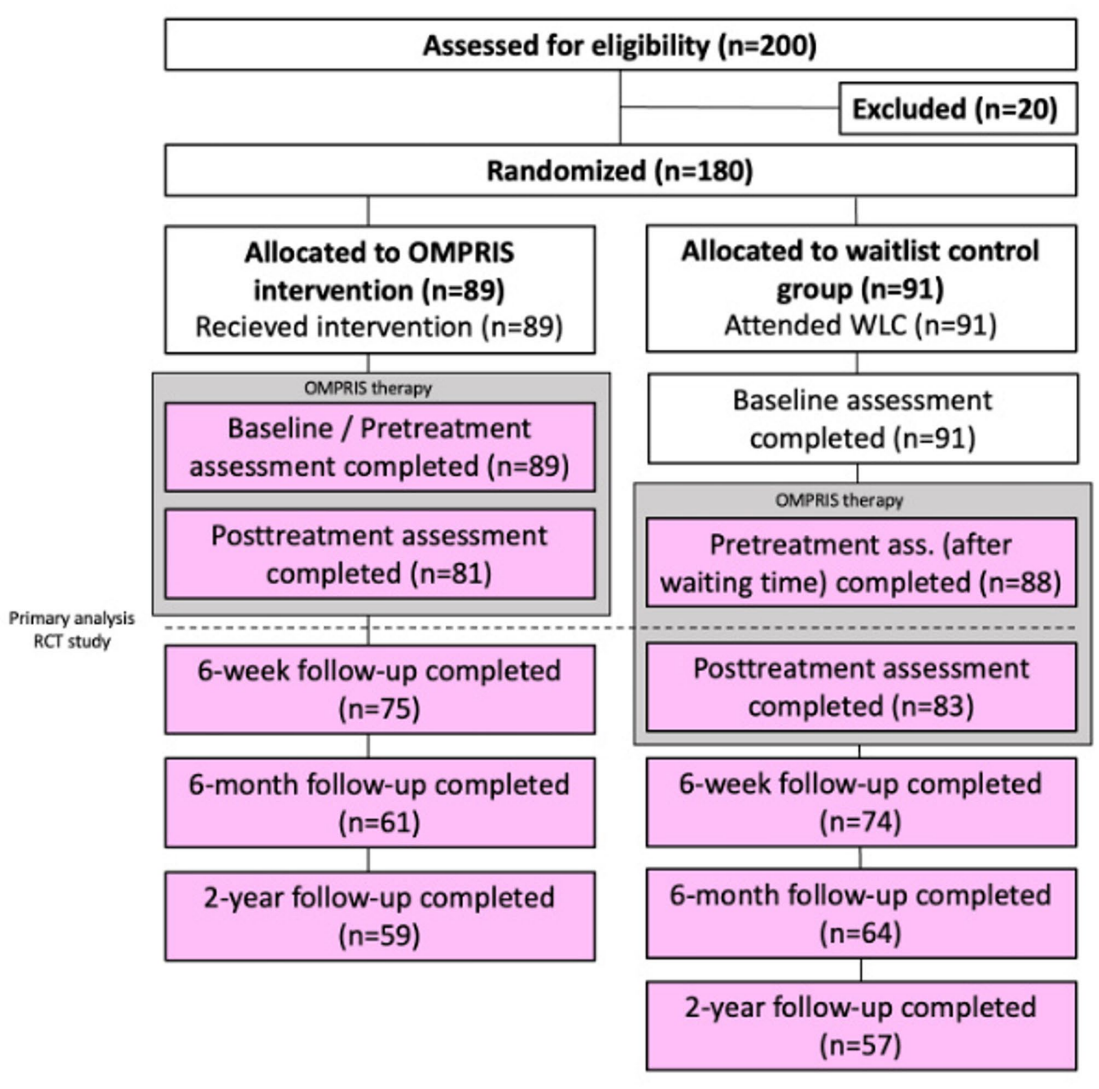
CONSORT Flowchart of the OMPRIS study. The gray boxes represent the period of the OMPRIS intervention. The pink boxes indicate which data were included in the 2-year follow-up analysis. For the waiting group, the assessment after completion of the waiting period was selected as pretreatment measurement.

This current 2-year follow-up study was a longitudinal observational study conducted with all participating IUD patients, regardless of whether they were in the control or treatment group. This procedure was chosen because the control group also received the intervention after the waiting period for ethical reasons. All 180 participants who were randomized at that time were contacted 2 years after the end of the intervention. We made contact via telephone, email, or mail. All data were collected via an online survey and were self-reported by the participants. Figure 1 shows the design of the original OMPRIS study and the data included in the analyses of this 2-year follow-up study (color-coded). For the waiting group, the assessment after completion of the waiting period was selected as pretreatment measurement.

### Assessment and instruments

#### IUD symptom severity

The primary outcome 2 years after the treatment was a reduction in IUD symptoms as measured by the Assessment of Internet and Computer Scale (AICA-S)^29^. The scale comprises 14 items on a 5-point Likert scale, aligning with the Diagnostic and Statistical Manual of Mental Disorders, Fifth Edition, criteria of substance use disorders and gambling disorders. These items include craving, loss of control, tolerance, unsuccessful attempts to reduce internet time, and withdrawal^30^. Furthermore, it assesses negative social consequences, time spent on the internet, and the preferred web-based activities. The cut-off is based on epidemiological surveys and analyses. A score of 7.0 to 13.0 points is rated as hazardous or moderately addictive internet use. A score of 13.5 points or more is considered pathological addictive internet use^31^. The questionnaire was developed and validated in Germany^29,32^. Validity and reliability of the AICA-S were checked in clinical and epidemiological surveys^32,33^. The internal consistency was found to be 0.83^29^.

#### Depression and anxiety symptoms

The Patient Health Questionnaire-9 (PHQ-9) and the Generalized Anxiety Disorder Screener (GAD-7) questionnaires, which are modules of the PHQ-D, were used to assess depression and anxiety^34–36^. Both the PHQ-9 and the GAD-7 were validated in Germany^37,38^. The PHQ-9 is a depression module that scores each of nine DSM-IV criteria as ‘0’(not at all) to ‘3’ (nearly every day). The internal consistency has been found to be excellent (*α* = 0.83–0.92)^37,39^. The GAD-7 scale is a self-report measure assessing general anxiety symptoms related to DSM-IV criteria on a 4-point Likert scale. The internal consistency of the GAD-7 scale has been demonstrated to be excellent, with a value of approximately 0.89–0.92^38,40^.

#### General life satisfaction

The short General Life Satisfaction scale was developed and validated in Germany and it^41^ (L-1) consists of only one item with the following wording: ‘How satisfied are you at present, all in all, with your life?’ The 11 answer categories of the L-1 range from ‘not satisfied at all’ to ‘completely satisfied’. The reliability has been tested by test–retest reliability, which has been reported to be *rtt* = 0.67^41^.

#### General internet use

We used the Compulsive Internet Use Scale (CIUS) to assess problematic internet use as a general construct^42^. In total, 14 items represent criteria for compulsive internet use. All items can be answered on a 5-point Likert scale, ranging from “never” (0) to “very often” (4). Participants scoring at least 28 points in the CIUS are at increased risk for pathological internet use. The CIUS has been successfully validated in Germany^43^. Good internal reliability criteria (Cronbach’s *α* ranging from 0.88 to 0.92) and a stable one-factor solution were found among different samples^42,43^.

#### Sociodemographic data

The following sociodemographic data were collected: age, gender, current marital status, housing situation, highest school-leaving qualification, vocational training, and current occupational situation.

#### Treatment after the OMPRIS intervention and satisfaction with OMPRIS

The number and type of treatments after the OMPRIS intervention were recorded. Furthermore, it was assessed if this treatment was provided on the recommendation of the OMPRIS consultants. In retrospect, satisfaction with the OMPRIS treatment was assessed on a scale of 0–10.

### Data analysis

All statistical analyses were performed in IBM SPSS Statistics (version 29.0; IBM Corp., Armonk, NY, USA) and R (version 4.2.1), and a two-sided significance level of 5% was used. Study characteristics were described using the mean ± standard deviation (SD) or proportions (%). A linear mixed-effects model (with random intercepts) using restricted maximum likelihood (REML) was performed to assess the association between the IUD symptom severity and the measurement time points (pretreatment, posttreatment, 6-week follow-up, 6-month follow-up, 2-year follow-up), controlling for the covariates age and gender. The model can be expressed as the following term: IUD ~ time + age + sex + (1 | id). The estimated marginal means of the linear mixed model (LMM) are given for the AICA-S scores. Time of measurement, age, and gender were selected as fixed effects, and the individual was selected as a random effect. Post-hoc tests were calculated using Bonferroni-correction. The estimated marginal mean is reported in the results. Further LMMs were calculated for all secondary outcomes using the same covariates. Furthermore, we used multiple linear regressions to estimate the association between the 2-year follow-up symptom severity (AICA-S score) as the dependent variable and the independent variables of AICA-S pre-treatment, time spent on the internet, AICA-S difference pre-post-treatment, PHQ-9 pre-treatment, GAD-7 pre-treatment, age, and treatment after intervention (y/n). Independent *t*-tests (2-sided) were used to compare age and baseline outcomes between 2-year follow-up responders and non-responders.

## Results

In total, data from 116 of 180 (64.4%) participants of the OMPRIS study were collected between September 2023 and April 2024. The 2-year follow-up sample included 25 women, 89 men, and 2 non-binary persons. They reported a mean age of 33.28 (SD = 11.28) years. All sociodemographic data are shown in (Table 1). A comparison of the baseline data between the patients who participated in the 2-year follow-up measurement and those who did not participate can be found in the appendix (suppl. Table S1). The 2-year follow-up non-responders were slightly older and reported slightly lower IUD symptom severity at baseline. The differences, however, did not reach statistical significance.

**Table 1:** Sociodemographic characteristics and current internet use.

	OMPRIS participants at 2-year follow-up (*N* = 116)
Socio-demographics	
Age, years	33.3 (11.3)
Gender	
Female	25 (22.6%)
Male	89 (76.7%)
Diverse	2 (1.7%)
Marital status	
Partnership	66 (56.9%)
No partnership	50 (43.1%)
Housing situation	
Living alone	32 (27.6%)
Living with partner	43 (37.1%)
Shared appartement	28 (24.1%)
Living in family	9 (7.8%)
Others	4 (3.4%)
School degree	
Low	1 (0.9%)
Middle	14 (12.1%)
High	101 (87.1%)
Highest level of professional training	
None	6 (5.2%)
Still undergoing vocational training	2 (1.7%)
Vocational training completed	17 (14.7%)
Currently still studying	35 (30.2%)
Successfully completed studies	54 (46.6%
Others	2 (1.7%)
Current job situation	
Unemployed	5 (4.3%)
Housewife / Househusband	2 (1.7%)
Primarily student or trainee	28 (24.1)
In work, part-time	24 (20.7%
In work, full-time	48 (41.4%
Retired	4 (3.4%)
Others	5 (4.3%)
Treatment situation	
Since OMPRIS participation in psychiatric treatment?	57 (49.1%)
Outpatient psychiatric care	13 (11.2%)
Outpatient psychotherapeutic treatment	43 (37.1%)
Outpatient addiction counseling	8 (6.9%)
Inpatient or day-care psychiatric / psychotherapeutic treatment	10 (8.6%)
Starting this treatment based on the recommendation by OMPRIS?	11 of 57 (19.3%)
Taking psychotropic medication	20 (17.2%)
Internet usage	
Internet usage 2 years after OMPRIS	
Internet usage weekdays (hrs./day)	3.8 (2.6)
Internet usage on weekends (hrs./day)	4.8 (3.1)
Weekly Internet usage (hrs./week), calculated	28.8 (18.4)
Internet application (most problematically)*	
Online streaming	47 (45.2%)
Online social media	18 (17.3%)
Online gaming	13 (12.5%)
Social pornography	12 (11.5%)
Others (e.g. online shopping, information research)	14 (13.5%)

*Based on 104 patients and 12 missings.

### Scale properties

Internal consistencies for all scales were acceptable to good. Specifically, Cronbach’s α was 0.74 for the AICA-S, 0.87 for the GAD-7, 0.81 for the PHQ-9, and 0.84 for the CIUS. As the L-1 consists of only one item, no Cronbach’s α was calculated.

### IUD Symptoms after two years

**Fig. 2:**
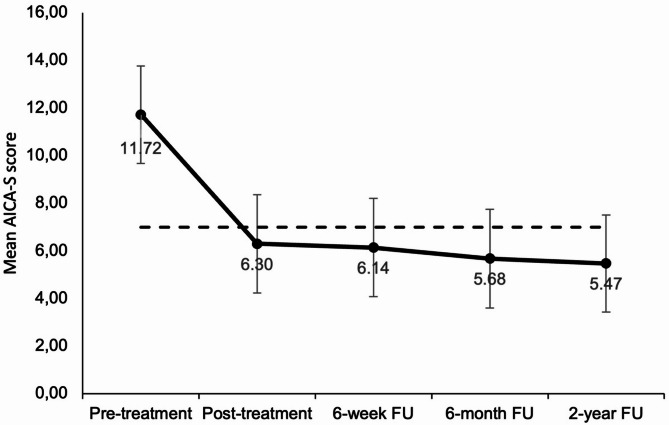
IUD symptom severity (AICA-S) at the five different measurement times of pre-treatment (T0), post-treatment (T2), 6-week follow-up (T3), 6-month follow-up (T4) and 2-year follow-up (T5).

The linear mixed model calculated a significant main effect of time (*p* < .001), while effects of gender (*p* = .856) and age (*p* = .526) did not reach significance. Figure 2; Table 2 provide the estimated marginal means for each measurement timepoint from T0 to T5. According to a post-hoc analysis with Bonferroni correction, the significant reduction in IUD symptom severity was only seen between pre-treatment and post-treatment measurements (*p* < .0001, estimated *M*_Diff_ = 5.418 points on the AICA-S scale). Post-treatment measurements and all follow-up measurements showed no significant differences (*p* = .632 to > 0.999, see suppl. Table S2 and S3). The results showed that there was no renewed increase in symptom severity after 2 years.

The data represent the estimated marginal means (error bars: 95% confidence interval) of the linear mixed model (LMM). The dashed line indicates the cut-off value for inconspicuous internet use (AICA-S cut-off 7.0).

**Table 2:** Descriptive analysis of pre-treatment (T0), post-treatment (T2), 6-week follow-up (T3), 6-month follow-up (T4) and 2-year follow-up (T5) measurements, estimated marginal means and change, results of the linear mixed models (LMMs).

	T0	T2	T3	T4	T5	Contrast T2-T5	*p* ^contrast^
Primary outcome score							
AICA-S	11.72 (1.03) [9.68, 13.76]	6.30 (1.04) [4.24, 8.36]	6.14 (1.04) [4.08, 8.20]	5.68 (1.05) [3.60, 7.75]	5.47 (1.03) [3.43, 7.51]	0.83 (0.44) [-0.43, 2.09]	0.632
Secondary outcome scores							
Internet usage (hrs./week)	46.1 (3.34) [39.5, 52.7]	29.3 (3.37) [22.7, 36.0]	28.9 (3.38) [22.2, 35.6]	29.6 (3.42) [22.9, 36.4]	28.8 (3.44) [22.1, 35.6]	0.49 (1.68) [-4.24, 5.23]	> 0.999
PHQ-9	10.21 (1.09) [8.06, 12.37]	8.29 (1.10) [6.11, 10.46]	8.12 (1.10) [5.94, 10.30]	7.84 (1.11) [5.65, 10.03]	7.39 (1.09) [5.23, 9.55]	0.89 (0.45) [-0.38, 2.18]	0.486
GAD-7	7.45 (0.99) [5.47, 9.42]	5.76 (1.00) [3.77, 7.75]	5.77 (1.00) [3.78, 7.77]	5.94 (1.01) [3.93, 7.94]	5.35 (1.00) [3.37, 7.33]	0.41 (0.40) [-0.74, 1.56]	> 0.999
CIUS	34.8 (2.38) [30.1, 39.5]	26.7 (2.40) [22.0, 31.5]	25.9 (2.40) [21.2, 30.7]	24.2 (2.41) [19.5, 29.0]	22.6 (2.38) [17.9, 27.3]	4.16 (0.93) [1.52, 6.81]	0.0001
L-1	5.04 (0.42) [4.20, 5.88]	6.32 (0.42) [5.47, 7.17]	6.04 (0.42) [5.19, 6.89]	6.29 (0.43) [5.44, 7.15]	6.59 (0.42) [5.75, 7.43]	-0.27 (0.19) [-0.80, 0.27]	> 0.999

Data are mean (SD) [95% CI] at pre-treatment (T0), post-treatment (T2), 6-week follow-up (T3), 6-month follow-up (T4) and 2-year follow-up (T5) periods.

*AICA-S* scale for the assessment of internet and computer game addiction, *PHQ-9* patient health questionnaire-9, *GAD-7* generalized anxiety disorder-7, *CIUS* compulsive internet use scale, *L-1* general life satisfaction short scale.

### Observed secondary outcomes after two years

**Fig. 3:**
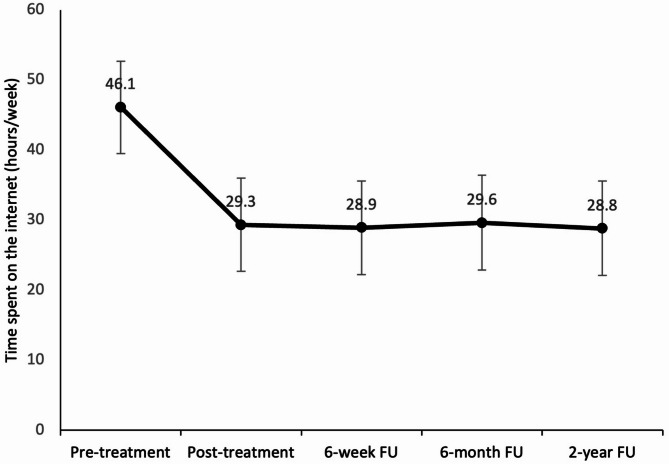
Time spent on the internet (hours/week) at the five different measurement times of pre-treatment (T0), post-treatment (T2), 6-week follow-up (T3), 6-month follow-up (T4) and 2-year follow-up (T5). The data represent the estimated marginal means (error bars: 95% confidence interval) of the linear mixed model (LMM).

The LMMs of the secondary outcomes, including the time spent on the internet (Fig. 3) and the CIUS, PHQ-9, GAD-7, and L-1 scores (suppl. Figure S1–S4 and suppl. Table S4-S8 in the appendix), revealed similar results. Table 2 includes the estimated marginal means for each measurement timepoint from T0 to T5 and each secondary outcome. While there was a significant decrease in symptoms and an increase in life satisfaction between pre-treatment and post-treatment (time spent on the internet: estimated M_Diff_ = 16.7 h/week, 95%-CI[12.63, 20.91], *p* < .0001; CIUS: M_Diff_ = 8.09, 95%-CI[5.46, 10.73], *p* < .0001; PHQ-9: M_Diff_ = 1.93, 95%-CI[0.65, 3.20], *p* = .003; GAD-7: M_Diff_ = 1.68, 95%-CI[0.54, 2.83], *p* = .0004; L-1: M_Diff_ = -1.28, 95%-CI[-1.82, -0.74], *p* < .0001), there were no significant differences between post-treatment and all follow-up measurements for PHQ-9, GAD-7, and time spent on the internet (*p* = .486 to > 0.999). We saw more significant drops in the CIUS score between post-treatment and 2-year follow-up (*p* = .0001), and between the 6-week follow-up and the 2-year follow-up (*p* = .0045). Again, gender and age were no significant covariates. All the exact values and figures for the LMMs can be found in the supplementary appendix.

### Descriptive analyses of IUD score differences in the 2-year follow-up

**Fig. 4:**
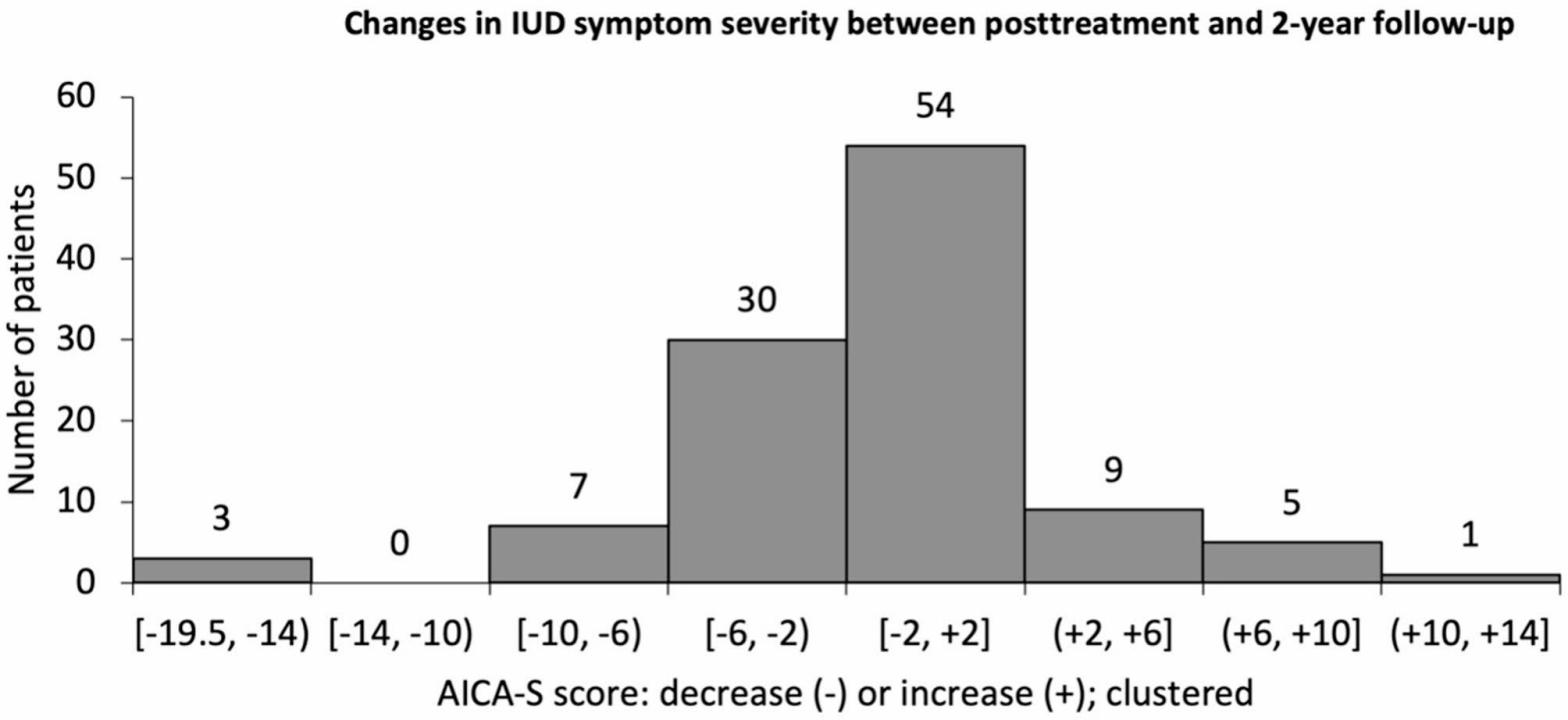
Frequency of increases or decreases in the AICA-S score compared post-treatment and 2-year follow-up assessment, *N* = 110. Round brackets mean an open interval, so the value at the bracket is not included, square brackets mean a closed interval, so the value at the bracket is included.

To examine the progression of individual IUD symptom severity in the post-treatment assessment, the differences in individual AICA-S scores between 2-year follow-up and post-treatment were calculated. The mean AICA-S difference was − 0.84 points (SD = 4.72, *n* = 110, range: -19.5 to 11.0 points). Overall, 32.7% (*n* = 36) of OMPRIS participants reported an increase in IUD symptoms after 2 years, while 58.2% (*n* = 64) of patients reported a further decrease in IUD symptoms. Furthermore, 9.1% of the patients (*n* = 10) reported no difference after 2 years. Figure 4 shows a histogram of the clustered frequencies of increases or decreases in the AICA-S score compared post-treatment and 2-year follow-up assessment.

### Predictors for IUD symptom severity in the 2-year follow-up

A multiple regression was conducted to find predictors for the AICA-S score in the 2-year follow-up measurement using the pre-treatment AICA-S score, difference between AICA-S pre- and post-treatment, pre-treatment time spent on the internet (weekdays), pre-treatment depression (PHQ-9) and anxiety (GAD-7) symptoms, age, and presence of further psychological treatment after intervention (n/y) as independent variables (Table 3). This resulted in a model, *F*(7, 108) = 6.456, *p* < .001, *R*^*2*^ = 0.309, adj. *R*^*2*^ = 0.261, which explained 30.9% of the variance in the 2-year AICA-S score. Higher pretreatment AICA-S scores (*B* = 0.557, *β* = 0.615, *t* = 4.65, *p* < .001) and a lower AICA-S reduction after the OMPRIS intervention (*B*=-0.441, *β*=-0.499, *t*=-5.129, *p* < .001) were significantly associated with higher AICA-S scores at the 2-year follow-up measurement. Pre-treatment time spent on the internet (weekdays), pre-treatment depression (PHQ-9), anxiety symptoms (GAD-7), and psychological treatment after the OMPRIS intervention declared no significant variance.

**Table 3:** Multiple linear regression to predict the AICA-S score after 2 years.

Dependent variable: AICA-S score 2-year follow-up							
Coefficient						95% CI	
	*B*	SE	*β*	*t*	*P*	*LL*	*UL*
(Constant)	3.753	1.669		2.249	0.027	0.442	7.064
AICA-S pretreatment	0.557	0.120	0.615	4.654	**< 0.001**	0.320	0.794
Time spent on the Internet	-0.128	0.121	-0.093	-1.053	0.295	-0.369	0.113
AICA-S difference pre-post	-0.441	0.086	-0.499	-5.129	**< 0.001**	-0.611	-0.270
PHQ-9 pretreatment	0.098	0.123	0.110	0.794	0.429	-0.147	0.343
GAD-7 pretreatment	-0.207	0.122	-0.224	-1.693	0.094	-0.449	0.035
Age	-0.033	0.033	-0.085	-1.002	0.318	-0.099	0.033
Treatment after OMPRIS (y/n)	0.215	0.766	0.024	0.280	0.780	-1.304	1.733

*N* = 109; *R*^*2*^ = 0.309; adj. *R*^*2*^ = 0.261; *F*(7,108) = 6.456; *p* < .001.

### Satisfaction with the OMPRIS intervention after 2 years

The mean satisfaction with OMPRIS two years after participation was still high (M = 7.26 of max. 10, SD = 2.11). The overall satisfaction with OMPRIS only slightly decreased two years after participation (post-treatment M = 8.60, SD = 1.33; 6-month follow-up M = 8.23, SD = 1.08).

## Discussion

The effectiveness of the OMPRIS intervention has already been demonstrated in an RCT design with a waiting control group^22^. It was also shown that there was no spontaneous relevant reduction of symptoms in the waiting group during the waiting period. The results of the long-term follow-up study successfully demonstrated that the reduction in symptom severity was still present after 2 years based on the mean value comparisons. On average, there was no relevant increase in IUD symptom severity after 2 years. On an individual level, it was shown that the symptom severity continued to decrease or remained stable for around 2/3 of the participants over the 2 years. In about 1/3 of the participants, the symptom burden increased again ( in most cases slightly) over the 2 years. This makes the OMPRIS study the first methodologically high-quality RCT study to prove the effect of online-based therapy via webcam in IUD patients not only in the short term^22^, but also over a period of two years.

Compared to previous studies for IUD treatment, our follow-up analysis is by far the longest to date. A systematic review from 2019 reports treatment effects of 12 CBT interventional studies across 6 different countries (efficacy in reducing IGD symptoms g = 0.92, [0.50,1.34]). Only six of these studies included a follow-up, with follow-ups occurring after 8 weeks, 12 weeks, 3 months, or 6 months^14^. A well-conducted RCT study from Germany published in 2019 at least carried out a follow-up measurement after 6 months^44^. Further high-quality recent meta-analyses of interventions unfortunately did not examine the presence of follow-up surveys in the included studies^8,45^. Compared to the existing literature, our study is thus the first RCT study to measure long-term follow-up after 2 years, which is an important indicator of the effectiveness of psychotherapy in IUD. From our point of view, it is essential to evaluate and assess the long-term effects of IUD interventions^25^.

In this study, two scales were used to assess the symptom severity of IUD. The AICA-S is a scale developed in Germany that allows a more differentiated clinical assessment. The evaluation is not just a sum score, but a differentiated assessment of each item, which makes it more rigorous and suited to identifying patients in a clinical setting. The AICA-S questionnaire has successfully been used as primary outcome in a comparable German trial in IUD patients treated with CBT group therapy^44^. In contrast, the CIUS has been used in numerous screening studies, which makes it internationally comparable as an well recognized questionnaire^46^. There is, however, a tendency for the CIUS to be very sensitive to IUD symptoms and to respond to them very quickly. To ensure a more stringent clinical diagnosis, we opted for the AICA-S as the primary outcome. However, *to* ensure good comparability with other international studies, we also assessed the CIUS as a secondary outcome. In our study, symptom reduction through the OMPRIS intervention was measured in both IUD scales used. While a clinically conspicuous value was measured before treatment, OMPRIS was able to reduce the symptom burden below the respective cut-off. The slightly more sensitive CIUS scale also showed an additional significant symptom reduction between post-treatment and 2-year follow-up (see supplementary data).

The psychological burden, such as symptoms of depression and anxiety, was assessed as a secondary outcome. The results showed that even after 2 years, there was no renewed increase in psychological burden. Recent meta-analyses have demonstrated that many intervention studies have found positive effects on, e.g., depression and anxiety, in addition to the reduction of IUD symptoms^8,45^. The results of the OMPRIS study therefore fit in well with the current state of knowledge. However, as described above, previous studies have only investigated up to a maximum of 6 months^14^. The OMPRIS intervention slightly reduced the depressive symptoms. While at baseline the participants exhibited “moderate depressive symptoms” on average (cut-off range: 10–14 points), after 2 years the value was in the “mild” range (< 10 points). For moderate depressive symptoms according to PHQ-9, the establishment of a treatment plan, intensive counseling, follow-up care and, if necessary, pharmacotherapy are recommended. In contrast, watchful waiting and repetition of the PHQ-9 during follow-up are recommended for mild depressive symptoms according to the PHQ-9. Regarding anxious symptoms measured with the GAD 7 score, the mean score remained in the same clinical category “mild anxiety” (cut-off range: 5–9 points). However, it should be noted that a proportion of participants underwent further outpatient treatment during the two years following OMPRIS. Theoretically, a more significant remission of depression and anxiety would have been desirable after two years. However, the symptoms scores were already only mild at baseline.

In particular, the IUD symptom severity at the beginning of therapy and a low decrease in the AICA-S score after the intervention were relevant prognostic factors for IUD symptom severity after 2 years. Both factors were associated with high AICA-S scores after 2 years. Depressive symptoms, anxiety symptoms, and age, on the other hand, had no significant influence on IUD symptom severity after 2 years, nor did the time spend on the internet at pretreatment assessment. Thus, the association appears to be IUD-specific and independent of the comorbidity of other mental disorders and age. On the one hand, the findings confirm other therapy studies that showed that a higher symptom burden before treatment was associated with a lower remission rate after treatment^44^. On the other hand, our results emphasize that the OMPRIS treatment was suitable for all age groups. Additionally, we assumed that further psychiatric treatment after completion of the OMRPIS intervention would have a positive effect on the course of symptoms. However, it was not a significant factor in our regression model. Clinicians typically recommend continuous psychotherapeutic coordinated care to prevent relapses in general addictions^47,48^, so this seems surprising. However, knowledge about predictors of treatment response in gaming disorders and IUD is still limited. Further studies are needed to identify positive predictors for treatment success with GD and IUD^18^.

OMPRIS did not lead to any negative consequences or side effects (e.g. an increase in depression or decrease of life satisfaction) and can therefore be integrated very well into other psychotherapeutic processes^22^. In addition, the majority of OMPRIS participants even described a higher level of satisfaction with the OMPRIS intervention even 2 years after participation. This shows that OMPRIS has made a lasting and meaningful impression on the participants and was really perceived as helpful. As a result of the COVID-19 pandemic and the necessary adaptations in the world of work, the use of webcam-based communication is now also accepted in psychotherapy^49,50^. Webcam-based therapy can now be offered to patients with IUD as an alternative to analog therapy if this is not desired or not possible due to life circumstances. With OMPRIS, we have created an effective treatment manual that therapists can easily integrate into their treatment planning.

This 2-year follow-up study has some limitations. Firstly, it must be said that 64 of 180 former randomized study participants could no longer be reached after 2 years. The reasons for this could be diverse. On the one hand, it could be explained by a change of contact information. On the other hand, it could also be that the more motivated participants were reached. This limitation must be taken into account in the interpretation. The analysis of the non-responders in relation to the baseline data, however, showed no significant difference. Secondly, symptom severity was assessed via a self-assessment rather than a clinical interview by a specialist, which was not possible due to the time involved for the participants and the investigators during the follow-up assessments. It can also be assumed that the greater time and effort for the participants would have resulted in an even higher drop-out rate. In addition, the use of validated questionnaires as an outcome measure in therapy studies is widespread and accepted. However, it would have been preferable to include an objective factor as an outcome, such as the automatically measured smartphone usage times. Thirdly, we no longer differentiated between the intervention group and the waiting group in this follow-up study. The consequence is that there is no longer a control group to control the group effect. This was no longer possible, as the waiting group also received the OMPRIS intervention after the waiting period. On the contrary, this even increases the number of responses after 2 years. Fourthly, it should also be noted that not only patients with severe symptoms took part in the OMPRIS study, but also people with mild IUD symptoms. OMPRIS should be both a preventive and a therapeutic offer. Therefore, the results can only be transferred to the clinical setting with some caution. However, a comparable therapy study, which was only conducted on clinical patients, showed an only slightly higher AICA-S baseline score^44^. Fifthly, it should be mentioned that the focus of the analysis was on the participants reached after 2 years. Therefore, no intent-to-treat analysis was conducted.

## Conclusions

The OMPRIS study contributes to the existing body of IUD treatment research by introducing for the first time a novel, telemedical, webcam-based treatment approach within a high-quality RCT design.

The OMPRIS participants demonstrated a significant reduction in IUD symptoms from pre- to post-treatment, with an effect size of *d* = 0.92^22^. In addition, this follow-up study showed that IUD symptom severity remained low even 2 years after intervention. This is the first time that the treatment effect of IUD therapy has been demonstrated in a long-term follow-up measurement of 2 years. Therefore, webcam-based, telemedical IUD therapy is an efficient treatment option that can now be offered for all patients who are unwilling or unable to undergo face-to-face psychotherapy.

## Electronic supplementary material

Below is the link to the electronic supplementary material.

Supplementary Material 1

## Data Availability

The datasets used and/or analyzed during the current study are available from the corresponding author on reasonable request.

## Abbreviations

AICA-S: Assessment of internet and computer scale
CIUS: Compulsive internet use scale
GAD-7: Generalized anxiety disorder screener - 7 items version
IUD: Internet use disorder
L-1: General life satisfaction scale
LMM: Linear mixed model
OMPRIS: Online-based motivational program to reduce problematic internet use and promote treatment motivation in gaming disorder and internet use disorders
PHQ-9: Patient health questionnaire - 9 items version
