# Supplementary material for: A 2-year follow-up analysis of individuals with internet use disorders treated with the webcam-based telemedicine OMPRIS intervention
Source: Sci Rep. 2025 Jul 31;15:27938. doi: 10.1038/s41598-025-12401-z (PMC12313996; doi:10.1038/s41598-025-12401-z)
Supplement: Supplementary file 1 — Supplementary Material 1 [file 41598_2025_12401_MOESM1_ESM.docx]

Supplementary Data

A two-year follow-up analysis of individuals with internet use disorders treated with the webcam-based telemedicine OMPRIS intervention.

Table of contents

[1. Comparison in OMPRIS participants between 2-year follow-up responder and non-responder 2](#_Toc179471756)

[2. Primary outcome AICS-S score, results from the linear mixed model, n=116. 3](#_Toc179471757)

[3. Secondary outcome: CIUS score, results of the linear mixed model, n=116. 4](#_Toc179471758)

[4. Secondary outcome: Time spent on the internet, results of the linear mixed model, n=116. 5](#_Toc179471759)

[5. Secondary outcome: PHQ-9 score, results of the linear mixed model, n=116. 6](#_Toc179471760)

[6. Secondary outcome: GAD-7 score, results of the linear mixed model, n=116. 7](#_Toc179471761)

[7. Secondary outcome: L-1 score, results of the linear mixed model, n=116. 8](#_Toc179471762)

## Comparison in OMPRIS participants between 2-year follow-up responder and non-responder

| **Suppl. Table S1: OMPRIS participants,** comparison between 2y-follow-up responder and non-responder | | |  |
| --- | --- | --- | --- |
|  | 2y follow-up responder | 2y follow-up non-responder |  |
|  | n=116 | n=61 | *p* |
| Age (y) | 30.59 (11.29) | 34.21 (13.68) | .061 |
| AICA-S baseline | 12.69 (6.63) | 11.57 (5.14) | .078 |
| PHQ-9 baseline | 10.18 (5.10) | 9.31 (4.94) | .274 |
| GAD-7 baseline | 7.18 (4.86) | 7.07 (4.53) | .875 |
| CIUS baseline | 35.46 (8.64) | 34.51 (7.96) | .466 |
| L-1 baseline | 4.82 (2.20) | 5.11 (2.16) | .394 |
| Time spent on the internet weekdays (h/d) | 6.17 (3.23) | 5.67 (3.44) | .351 |
| Time spent on the internet weekend (h/d) | 6.66 (3.03) | 6.57 (3.52) | .859 |
| *Notes.* Two-sides p, *t*-tests for equality of means | |  |  |

Table S1 shows that non-responders were slightly older and reported slightly lower IUD symptom severity at baseline. The differences, however, did not reach statistical signification. All other outcomes showed non-significant differences.

## Primary outcome AICS-S score, results from the linear mixed model, n=116.

Suppl. Table S2: Results of the linear mixed model, n=116.

| Fixed Effects | | | | | | | |
| --- | --- | --- | --- | --- | --- | --- | --- |
|  | Effect | Est/Beta | SE | 95% CI | | t | p |
| Intercept |  | 11.71 | 1.03 | 9.69 – 13.74 | | 11.35 | <.001 |
| Time1 | 2-1 | 6.30 | 1.04 | 4.26 - 6.27 | | 6.05 | <.001 |
| Time2 | 3-1 | 6.13 | 1.04 | 4.09 – 8.18 | | 5.88 | <.001 |
| Time3 | 4-1 | 5.67 | 1.04 | 3.62 – 7.73 | | 5.41 | <.001 |
| Time4 | 5-1 | 5.47 | 1.03 | 3.44 – 7.49 | | 5.29 | <.001 |
| Sex1 | F-M | -0.41 | 0.87 | -2.13 – 1.29 | | -0.48 | .633 |
| Sex2 | D-M | -0.92 | 2.89 | -6.60 – 4.74 | | -0.32 | .749 |
| Age | Age | -0.02 | 0.03 | -0.08 – 0.04 | | -0.63 | .526 |
| Random Effects | | | | | | | |
|  | | | | Variance | | S.D. | Correlation |
| Participant (Intercept) | | | | 12.6 | | 3.55 | 0.531 |
| Residual | | | | 11.1 | | 3.33 |  |
| Model fit | | | | | | | |
| R^2^ | | | | | Marginal | Conditional | |
|  | | | | | 0.197 | 0.624 | |
| Note. Linear mixed model fit by REML, Number of observations: 545, group: ID=116, Model equation: AICA-S ~ Time + Sex + Age +(1 \| ID). F=Female, M=Male, D=Divers | | | | | | | |

| Suppl. Table S3: Pairwise Comparisons between assessment time points, post-hoc tests | | | | | | |
| --- | --- | --- | --- | --- | --- | --- |
| (I) Time | (J) Time | Estim. mean diff. (I-J) | Std. error | *P*_bonf._ | 95% Confidence Interval for Difference | |
|  |  |  |  |  | Lower Bound | Upper Bound |
| pre | post | 5.418* | 0.444 | <.0001 | 4.16 | 6.67 |
|  | 6 weeks | 5.582* | 0.449 | <.0001 | 4.31 | 6.85 |
|  | 6 months | 6.042* | 0.466 | <.0001 | 4.72 | 7.36 |
|  | 2 years | 6.248* | 0.441 | <.0001 | 5.00 | 7.49 |
| post | 6 weeks | 0.164 | 0.451 | 1.000 | -1.11 | 1.44 |
|  | 6 months | 0.624 | 0.468 | 1.000 | -0.69 | 1.94 |
|  | 2 years | 0.830 | 0.45 | 0.632 | -0.43 | 2.09 |
| 6 weeks | 6 months | 0.460 | 0.471 | 1.000 | -0.87 | 1.79 |
|  | 2 years | 0.666 | 0.450 | 1.000 | -0.61 | 1.94 |
| 6 months | 2 years | 0.666 | 0.450 | 1.000 | -1.12 | 1.52 |
| *Notes.* Based on estimated marginal means. Results are averaged over the levels of: Sex, Degrees-of-freedom method: Kenward-Roger, P value adjustment: Bonferroni method for 10 tests, * The estimated mean difference is significant at the .05 level. | | | | | | |

## Secondary outcome: CIUS score, results of the linear mixed model, n=116.

Suppl. Table S4: Results of the linear mixed model, n=116.

| Fixed Effects | | | | | | | |
| --- | --- | --- | --- | --- | --- | --- | --- |
|  | Effect | Est/Beta | SE | 95% CI | | t | p |
| Intercept |  | 26.86 | 2.32 | 22.33 – 31.41 | | 11.59 | <.001 |
| Time1 | 2-1 | -8.09 | 0.94 | -9.93 - -6.27 | | -8.66 | <.001 |
| Time2 | 3-1 | -8.89 | 0.98 | -10.75 - -7.04 | | -9.38 | <.001 |
| Time3 | 4-1 | -10.61 | 0.98 | -12.52 - -8.68 | | -10.81 | <.001 |
| Time4 | 5-1 | -12.26 | 0.93 | -14.08 - -10.44 | | -13.21 | <.001 |
| Sex1 | F-M | -0.24 | 2.04 | -4.25 – 3.76 | | -0.12 | .905 |
| Sex2 | D-M | -0.48 | 6.70 | -13.63 – 12.66 | | -0.07 | .942 |
| Age | Age | -0.13 | 0.07 | -0.28 – 0.02 | | -1.71 | .090 |
| Random Effects | | | | | | | |
|  | | | | Variance | | S.D. | Correlation |
| Participant (Intercept) | | | | 71.0 | | 8.43 | 0.591 |
| Residual | | | | 49.2 | | 7.01 |  |
| Model fit | | | | | | | |
| R^2^ | | | | | Marginal | Conditional | |
|  | | | | | 0.148 | 0.651 | |
| Note. Linear mixed model fit by REML, Number of observations: 544, group: ID=116, Model equation: Cius ~ Time + Sex + Age +(1 \| ID). F=Female, M=Male, D=Divers | | | | | | | |

| **Suppl. Figure S1.** Mean CIUS score at the five different measurement times of pre-treatment (T0), post-treatment (T2), 6-week follow-up (T3), 6-month follow-up (T4) and 2-year follow-up (T5). Data are estimated marginal means (error bars: 95%-Confidence Interval) of the linear mixed model (LMM). |
| --- |

## Secondary outcome: Time spent on the internet, results of the linear mixed model, n=116.

Suppl. Table S5: Results of the linear mixed model, n=116.

| Fixed Effects | | | | | | | |
| --- | --- | --- | --- | --- | --- | --- | --- |
|  | Effect | Est/Beta | SE | 95% CI | | t | p |
| Intercept |  | 32.55 | 3.23 | 26.2 – 38.8 | | 10.07 | <.001 |
| Time1 | 2-1 | -16.76 | 1.45 | -19.6 - -13.8 | | -11.41 | <.001 |
| Time2 | 3-1 | -17.20 | 1.51 | -20.2 - -14.23 | | -11.33 | <.001 |
| Time3 | 4-1 | -16.48 | 1.60 | -19.6 - -13.3 | | -10.28 | <.001 |
| Time4 | 5-1 | -17.25 | 1.66 | -20.5 - -14.0 | | -10.39 | <.001 |
| Sex1 | F-M | -3.45 | 2.81 | -8.9 – 2.0 | | -1.22 | .221 |
| Sex2 | D-M | 0.06 | 9.37 | -18.3 – 18.4 | | -0.00 | .995 |
| Age | Age | -.033 | 0.09 | -0.5 – -0.1 | | -3.41 | .<.001 |
| Random Effects | | | | | | | |
|  | | | | Variance | | S.D. | Correlation |
| Participant (Intercept) | | | | 201 | | 14.2 | 0.527 |
| Residual | | | | 180 | | 13.4 |  |
| Model fit | | | | | | | |
| R^2^ | | | | | Marginal | Conditional | |
|  | | | | | 0.155 | 0.600 | |
| Note. Linear mixed model fit by REML, Number of observations: 544, group: ID=116, Model equation: Use ~ Time + Sex + Age +(1 \| ID). F=Female, M=Male, D=Divers | | | | | | | |

## Secondary outcome: PHQ-9 score, results of the linear mixed model, n=116.

Suppl. Table S6: Results of the linear mixed model, n=116.

| Fixed Effects | | | | | | | |
| --- | --- | --- | --- | --- | --- | --- | --- |
|  | Effect | Est/Beta | SE | 95% CI | | t | p |
| Intercept |  | 8.37 | 1.05 | 6.29 – 10.45 | | 7.90 | <.001 |
| Time1 | 2-1 | -1.92 | 0.45 | -2.81 - -1.03 | | -4.25 | <.001 |
| Time2 | 3-1 | -2.09 | 0.45 | -2.99 - -1.18 | | -4.45 | <.001 |
| Time3 | 4-1 | -2.37 | 0.47 | -3.30 - -1.44 | | -4.99 | <.001 |
| Time4 | 5-1 | -2.82 | 0.45 | -3.70 - -1.94 | | -6.27 | <.001 |
| Sex1 | F-M | 0.61 | 0.93 | -1.20 – 2.44 | | 0.66 | .508 |
| Sex2 | D-M | 0.54 | 3.01 | -5.46 – 6.55 | | 0.178 | .859 |
| Age | Age | 0.007 | 0.03 | -0.05 – 0.07 | | 0.22 | .819 |
| Random Effects | | | | | | | |
|  | | | | Variance | | S.D. | Correlation |
| Participant (Intercept) | | | | 14.4 | | 3.8 | 0.555 |
| Residual | | | | 11.6 | | 3.4 |  |
| Model fit | | | | | | | |
| R^2^ | | | | | Marginal | Conditional | |
|  | | | | | 0.039 | 0.572 | |
| Note. Linear mixed model fit by REML, Number of observations: 544, group: ID=116, Model equation: PHQ ~ Time + Sex + Age +(1 \| ID). F=Female, M=Male, D=Divers | | | | | | | |

**Suppl. Figure S2.** Mean PHQ-9 score at the five different measurement times of pre-treatment (T0), post-treatment (T2), 6-week follow-up (T3), 6-month follow-up (T4) and 2-year follow-up (T5). Data are estimated marginal means (error bars: 95%-Confidence Interval) of the linear mixed model (LMM).

## Secondary outcome: GAD-7 score, results of the linear mixed model, n=116.

Suppl. Table S7: Results of the linear mixed model, n=116.

| Fixed Effects | | | | | | | |
| --- | --- | --- | --- | --- | --- | --- | --- |
|  | Effect | Est/Beta | SE | 95% CI | | t | p |
| Intercept |  | 6.05 | 0.97 | 4.15 – 7.95 | | 6.23 | <.001 |
| Time1 | 2-1 | -1.68 | 0.40 | -2.47 - -0.88 | | -4.14 | <.001 |
| Time2 | 3-1 | -1.67 | 0.41 | -2.48 - -0.86 | | -4.06 | <.001 |
| Time3 | 4-1 | -1.50 | 0.42 | -2.34 - -0.67 | | -3.53 | <.001 |
| Time4 | 5-1 | -2.09 | 0.40 | -2.88 - -1.30 | | -5.19 | <.001 |
| Sex1 | F-M | 0.29 | 0.85 | -1.37 – 1.97 | | 0.34 | .730 |
| Sex2 | D-M | 0.35 | 2.81 | -5.15 – 5.85 | | 0.12 | .901 |
| Age | Age | -0.001 | 0.03 | -0.06 – 0.06 | | -0.04 | .968 |
| Random Effects | | | | | | | |
|  | | | | Variance | | S.D. | Correlation |
| Participant (Intercept) | | | | 12.25 | | 3.50 | 0.558 |
| Residual | | | | 9.30 | | 3.05 |  |
| Model fit | | | | | | | |
| R^2^ | | | | | Marginal | Conditional | |
|  | | | | | 0.0256 | 0.5795 | |
| Note. Linear mixed model fit by REML, Number of observations: 544, group: ID=116, Model equation: GAD ~ Time + Sex + Age +(1 \| ID). F=Female, M=Male, D=Divers | | | | | | | |

**Suppl. Figure S3.** Mean GAD-7 score at the five different measurement times of pre-treatment (T0), post-treatment (T2), 6-week follow-up (T3), 6-month follow-up (T4) and 2-year follow-up (T5). Data are estimated marginal means (error bars: 95%-Confidence Interval) of the linear mixed model (LMM).

## Secondary outcome: L-1 score, results of the linear mixed model, n=116.

Suppl. Table S8: Results of the linear mixed model, n=116.

| Fixed Effects | | | | | | | |
| --- | --- | --- | --- | --- | --- | --- | --- |
|  | Effect | Est/Beta | SE | 95% CI | | t | p |
| Intercept |  | 6.05 | 0.40 | 5.25 – 6.85 | | 14.78 | <.001 |
| Time1 | 2-1 | 1.27 | 0.19 | 0.90 – 1.65 | | 6.73 | <.001 |
| Time2 | 3-1 | 0.99 | 0.19 | 0.62 – 1.37 | | 5.18 | <.001 |
| Time3 | 4-1 | 1.25 | 0.19 | 0.86 – 1.64 | | 6.29 | <.001 |
| Time4 | 5-1 | 1.55 | 0.18 | 1.18 – 1.92 | | 8.21 | <.001 |
| Sex1 | F-M | 0.16 | 0.35 | -0.53 – 0.86 | | 0.45 | .651 |
| Sex2 | D-M | -0.007 | 1.18 | -2.33 – 2.31 | | -0.006 | .995 |
| Age | Age | -0.02 | 0.01 | -0.04 – 0.008 | | -1.23 | .200 |
| Random Effects | | | | | | | |
|  | | | | Variance | | S.D. | Correlation |
| Participant (Intercept) | | | | 14.4 | | 3.8 | 0.555 |
| Residual | | | | 11.6 | | 3.4 |  |
| Model fit | | | | | | | |
| R^2^ | | | | | Marginal | Conditional | |
|  | | | | | 0.077 | 0.542 | |
| Note. Linear mixed model fit by REML, Number of observations: 544, group: ID=116, Model equation: L1 ~ Time + Sex + Age +(1 \| ID). F=Female, M=Male, D=Divers | | | | | | | |

**Suppl. Figure S4.** Mean L-1 score at the five different measurement times of pre-treatment (T0), post-treatment (T2), 6-week follow-up (T3), 6-month follow-up (T4) and 2-year follow-up (T5). Data are estimated marginal means (error bars: 95%-Confidence Interval) of the linear mixed model (LMM).
